# Efficacy and safety of remimazolam in procedural sedation and analgesia: A protocol for systematic review and meta analysis

**DOI:** 10.1097/MD.0000000000020765

**Published:** 2020-07-02

**Authors:** Feng Wang, Qian Zhou, Minhuan Shen, Jing Quan, Jiejuan Chen, Jing Shi, Xiaohua Zou

**Affiliations:** Department of Anesthesiology, Affiliated Hospital of Guizhou Medical University, Gui yang, China.

**Keywords:** efficacy and safety, endoscopy, meta-analysis, midazolam, remimazolam

## Abstract

**Background:**

Remimazolam is a newly developed benzodiazepine as an alternative of conventional sedatives in the procedure of anesthesia. For the purpose of evaluating the efficacy and safety of remimazolam sedation during an endoscopy, we will perform a systematic review and meta-analysis of randomized controlled trials that compared remimazolam with midazolam and/or placebo.

**Methods:**

We will search PubMed, Embase, Web of Science, and the Cochrane Controlled Register of Trials (CENTRAL) from inception to December 2019 for randomized controlled trials that investigated efficacy and safety of remimazolam during an endoscopy. The job will be performed without language restriction. Experimental groups will include the interventions of remimazolam, while control groups will involve midazolam, placebo, or no controls. The primary outcome will be the onset time, followed by the secondary outcomes of the recovery time, the incidence of hypotension, the incidence of hypoxia and the incidence of bradycardia. Relative ratio or standardized mean difference will be used to measure the effect size of remimazolam. We will use *I*^2^ statistics to assess the between-study heterogeneity in each meta-analysis, Eager's test to detect publication bias.

**Results:**

The results of this study will be published in a peer-reviewed journal.

**Ethics and dissemination:**

There is no need for ethical approval because all data used in this meta-analysis have been published. In addition, all data will be analyzed anonymously during the review process.

**Protocol registration number:**

CRD42020170745.

## 1 Introduction

Endoscopy, including gastroscopy, colonoscopy, bronchoscopy, and other types of endoscopy, is usually used to investigate suspected lesions and conduct biopsy or certain surgeries. An endoscopic procedure is uncomfortable and restless, although not painful, for most patients who may be given a local anesthetic or offered a certain sedative. To satisfy the demands of higher efficiency and shorter hospital stay, endoscopists often choose the sedative with characteristics of short recovery time, few accumulation effects and fast neuropsychic function recovery.^[1,2]^

Midazolam, as a commonly used drug of benzodiazepine, expressed good performances of amnesia, titration and applicability. Although many endoscopists prefer to use it during endoscopic procedures, midazolam has some obvious disadvantages. Long-acting metabolite (with an elimination half-life of 1.8–6.4 h) leads to slow recovery of neuropsychic function, causing greater accumulation effects than its counterparts of the propofol class. Rather, the optimal dose of Midazolam differs among subjects, triggering prolonged sedation and even respiratory depression or failure to achieve effective sedative state.^[3,4]^ Early randomized controlled trials have identified that volunteers recovered faster from remimazolam than midazolam in procedural sedation, followed by subsequent evaluations about the efficacy and safety of remimazolam in cardiac surgery and colonoscopy relative to Midazolam. However, existing reports have not sufficiently presented meaningful differences between remimazolam and its counterparts because the number of enrolled subjects is limited.^[5–7]^ Therefore, we aimed to develop study protocol of a systematic review and meta-analysis, clarifying whether remimazolam is at least as effective as midazolam but with less adverse effects and whether remiazolam is superior over placebo in the management of anesthesia during an endoscopy.

## 2 Methods

### 1.1 Study registration

We develop the protocol in adherence to Preferred Reporting Items for Systematic Review and Meta-Analysis Protocols (PRISMA-P),^[8]^ and it protocol has been registered on PROSPERO (https://www.crd.york.ac.uk/prospero) with a unique ID of CRD42020170745.

### 1.2 Data sources and search strategy

We will search OVID Medline, Embase, and the Cochrane Controlled Register of Trials (CENTRAL) from inception to June 1, 2020 for RCTs that tested the efficacy and safety of remimazolam for an endoscopy. Comprehensive search strategy will be developed by combining medical subject headings and keywords: “remimazolam,” “midazolam,” and “endoscopy.” Articles published in any language will be included. The reference lists of the identified studies will be manually searched for any missing RCTs.

### 1.3 Eligibility criteria and study selection

We will include participants undergoing endoscopic procedures, including gastrointestinal endoscopy, colonoscopy, gastrointestinal endoscopy, and bronchoscopy. We will screen for studies that evaluated the efficacy and safety of remimazolam by comparing it with midazolam and/or placebo. Studies with any of the following conditions will be excluded:

1. the included participants undergoing any endoscopic procedures with unclear anesthetics;
2. compared with any other anesthetics in the control group instead of midazolam;
3. data are unavailable or even incorrect, or without any relevant data for meta-analysis;
4. duplicated publications.

Two reviewers will screen the articles according to the eligibility criteria; one will screen the titles, abstracts, and full-text of the articles, and the other one will check the results. Disagreement between the two reviewers will be solved by group discussion.

### 1.4 Outcome measurements

The primary outcome will be the onset time, while the second outcomes will be the recovery time, the incidence of hypotension, the incidence of hypoxia, and the incidence of bradycardia.

### 1.5 Data extraction

Data extraction would include the baseline of the study (first author, publication year, country, number, gender and age of the participant, and the procedure), the specific interventions they received (name, dose, frequency, and the total duration of treatment), the monitoring for efficacy or adherence, and the measure of outcome (specifically defined as event or measure and time frame for the ascertainment of this outcome). For studies with missing data, we will attempt to contact the corresponding authors for details.

Two reviewers will extract data; one will extract the aforementioned information, and the other one will check the completeness and correctness of the extracted information.

### 1.6 Risk of bias assessment

The Cochrane Collaboration tool for assessing the risk of bias (ROB) would be used to appraise the methodological quality of the included studies by two reviewers independently. If necessary, the corresponding author would arbitrate any discrepancies between the two reviewers to avoid the bias.

### 1.7 Data synthesis

We will calculate the effect size in contrast to midazolam and/or placebo of remimazolam in each study; continuous data will be calculated by using standardized mean difference (SMD) and dichotomous data using relative ratio (RR). We will use Cochran's *Q* test to examine whether there is significant between-study heterogeneity, and we will classify the degree of heterogeneity by using *I*^2^ statistics—small heterogeneity (*I*^2^ < 50%), large (50% ≤ *I*^2^ < 75%), and very large (*I*^2^ ≥ 75%). Meta-analysis will be performed with fixed-effect model when there is insignificant heterogeneity, otherwise it will be performed with random-effect model.

## 3 Discussion

Remimazolam is a carboxylate derivative of benzodiazepines, possessing a high affinity for benzodiazepine receptors in cerebral cortex, limbic system, midbrain and brainstem spinal cord. It can promote the binding of the GABA receptors, increasing the frequency of the CI-channel opening, thus triggering hyperpolarization of nerve cells and finally producing the effect of neuroinhibitory.^[9,10]^ Generally, the context-sensitive half-time of conventional sedatives in intravenous infusion may increase as a result of the extending of infusion time, triggering the slowing down of metabolism in vivo. Meanwhile, through animal testing, Upton et al reported that the context-sensitive half-time of remimazolam varied slightly when the infusion time extended, clarifying that the metabolism in vivo is more active and thus having a shorter action time.^[11,12]^ After different doses of remimazolam were given, although the respiratory rate and PaO_2_ of the animals decreased temporarily, there were no obvious changes upon PaCO_2_, SaO_2_, mean arterial blood pressure, heart rate, and central venous pressure.^[12]^ As far as we know, many researchers have been reported about the efficacy and safety of remimazolam.^[13–16]^ However, many drawbacks in the design and implementation of some randomized controlled trials of remimazolam-related researches exist. Any neglect of every process may lead to bias, affecting the effect of the evaluation. This review compared remimazolam with midazolam and/or placebo as to the onset time and the recovery time. In addition, we would report related adverse events about hypotension, hypoxia, and bradycardia. It is expected that this systematic review and meta-analysis will encourage further research to furnish high-quality evidence which is currently insufficient or lacking.

## Author contributions

Feng Wang and Qian Zhou Conceived the idea for this study; Xiaohua Zou Provided statistical advice and input; Qian Zhou, Minhuan Shen drafted the study. Jing Quan, Jiejuan Chen, Jing Shi reviewed the study and provided critical feedback.

All authors approved the article in its final form.
